# Inflammatory Markers as Predictors for Prolonged Duration of Hospitalization in Maxillofacial Infections

**DOI:** 10.3390/jcm12030871

**Published:** 2023-01-21

**Authors:** Horatiu Urechescu, Eleonora Gheran-Vida, Cristiana Cuzic, Oana Ancusa, Sorin Ursoniu, Marius Pricop

**Affiliations:** 1 Discipline of Oral and Maxillo-Facial Surgery, Faculty of Dental Medicine, “Victor Babes” University of Medicine and Pharmacy Timisoara, Eftimie Murgu Square 2, 300041 Timisoara, Romania; 2 Timisoara Emergency Clinical Municipal Hospital, Hector 1 Street, 300041 Timisoara, Romania; 3 Department of Prosthodontics, Faculty of Dental Medicine, “Victor Babes” University of Medicine and Pharmacy, Eftimie Murgu Square 2, 300041 Timisoara, Romania; 4 Department V, Discipline of Medical Semiology I, Faculty of General Medicine, “Victor Babes” University of Medicine and Pharmacy Timisoara, Eftimie Murgu Square 2, 300041 Timisoara, Romania; 5 Department of Functional Sciences, Center for Translational Research and Systems Medicine, “Victor Babes” University of Medicine and Pharmacy, Eftimie Murgu Square 2, 300041 Timisoara, Romania

**Keywords:** health care services, C-reactive protein (CRP), hemogram indexes, maxillofacial infection (MFI), white blood cell count (WBC)

## Abstract

Despite the progress made in diagnosing and treating maxillofacial infections, the course of infection can be unpredictable, leading to severe complications, prolonged hospitalization, and substantial financial costs to health care services. It is important to determine whether various serum inflammatory marker levels on admission may predict a prolonged hospital stay in these patients. To analyze the role of CRP, white blood cell count (WBC), and neutrophil-to-lymphocyte ratio (NLR) in predicting the prolonged duration of hospitalization in maxillofacial infections, we performed a retrospective study by collecting paper records data from 108 patients who met our inclusion criteria. The patients were divided into two groups according to the duration of hospitalization (group A < 5 days and group B ≥ 5 days). The predictor variables were CRP, WBC, and NLR, and the outcome variable was the duration of hospitalization. This study confirmed a positive linear correlation (*p* < 0.001) between the predictors and the outcome variable. The optimal cut-off values for WBC are 11,030 white blood cells/μL and 63 mg/L for CRP. Levels that exceed these optimal values predict a duration of hospitalization of over (≥) 5 days. Serum WBC and CRP on admission may predict the duration of hospitalization in patients with MFI.

## 1. Introduction

Acute maxillofacial infections (MFIs) affect a significant part of the population and are among the most common conditions treated in the maxillofacial department [1]. Despite the progress made in diagnosing and treating the disease, even today, the course of infection can be unpredictable. It can lead to severe complications causing extensive mortality and morbidity [2,3] associated with a mortality rate of 10–40% [4]. Maxillofacial infections usually occur in the fascial planes and are self-limiting [5,6]. Due to the complex anatomical characteristics of fascial spaces that are connected to each other, the propagation of the infections into deep fascial spaces can be produced by direct continuity by lymphatic or hematogenous dissemination and depends on the patient’s local and systemic factors, as well as on the virulence of the pathogen [7], leading to fatal consequences. Multiple life-threatening complications have been reported, such as airway obstruction [1,6,8], necrotizing fasciitis [9,10], descending mediastinitis [11,12], thoracic empyema [13,14], cavernous sinus thrombosis [15,16], cerebral abscess [17,18], mandibular or cervical osteomyelitis [19,20], and sepsis [21,22] with disseminated intravascular coagulation [23]. Early assessment of the prognostic, timely, and effective treatment is essential for faster recovery, shorter hospitalization, and reduced risk of severe systemic complications [24]. In these patients, a prolonged hospital stay is associated with substantial financial costs to health care services. The “faster recovery” describes a patient whose medical condition progresses to an improved condition, demonstrating the efficacy of the treatment, at a rate that is below the average. In contrast, the term “prolonged hospitalization” describes a patient whose improved medical condition is progressing at a rate that is above the average. This could be due to a number of different factors, such as age, state of health, or the severity of the infection.

In various published research, the criteria for prolonged hospitalization differ between studies. Usually, hospitalization over the average period is considered long-term admission. In the USA, the average length of stay was 3 to 8.3 days [25,26,27,28]; in Iran, it was 6.8 days [29]; in Finland, it was 14.8 days [30]; in China, it was 12 days [31]; and in Romania, it was 4.92 days [32]. From the SCMUT database, the median LOS of the inpatients in our unit in the last 5 years was 5.5 days [33]. This indicates that the length of hospitalization is different in different regions of the world when similar adult infections are compared; however, the number of studies comparing hospitalization length among different countries is too low to make an accurate comparison.

Patients generally remain hospitalized until the infection resolves or is controlled, and until the patient returns to a pre-infection state of health. 

Furthermore, the average length of stay (LOS) of maxillofacial infection patients varies and is usually affected by multiple confounding variables such as the patient’s medical status, the severity of the infection, differences in antibiotic therapy, timelines of surgical intervention in the form of incision and drainage of infected spaces, and the presence or absence of underlying disease that may affect the outcome [34]. Much of the published evidence identified that age, preadmission antibiotic use [28], underlying co-morbidities and ASA (American Society of Anesthesiology) score classification [35], diabetes mellitus [34], higher odontogenic infection severity score [28], the number of infected spaces, and infection site [36] were associated with increased LOS.

Finally, the length of hospital stay may be affected by financial factors. There is a difference in the cost of hospitalization because the health insurance system is different in each country [26]. In the USA, daily mean room and bed charges range from USD 978 to USD 1598 [28]; on the other hand, in Romania, they range from USD 76.79 to USD 197.49 per day [37] if the patient benefits from health insurance. In our study, the average cost per night for a bed in the Department of Maxillofacial Surgery of SCMUT hospital is USD 197.69 (RON 900). When day surgeries were added, the average cost per patient with maxillofacial infection was USD 389.28 (RON 1773).

It is therefore important to determine whether various serum inflammatory marker levels on admission may predict a prolonged hospital stay in patients with acute maxillofacial infections. Among the most commonly evaluated inflammatory markers, the C-reactive protein (CRP), white blood cell count, and hemogram indexes provide valuable information to clinicians for the diagnosis, screening, and follow-up of various diseases [38].

Therefore, this study was performed to analyze the role of CRP, white blood cell count (WBC), and neutrophil-to-lymphocyte ratio (NLR) in predicting the prolonged duration of hospitalization in adult patients with maxillofacial infections. The null hypothesis of this study is that WBC, CRP, and NLR values do not change the variability of the duration of hospital stay in these patients.

## 2. Materials and Methods

### 2.1. Study Design and Patient Selection Process

We performed a retrospective study on patients hospitalized at the Department of Maxillofacial Surgery—City Emergency Hospital Timisoara (SCMUT)—affiliated with the Victor Babes University of Medicine and Pharmacy Timisoara for acute maxillofacial infections. From a total of 544 patients treated for maxillofacial conditions in our department between January 2017 and April 2022, 108 (89.2%) met our inclusion criteria and were subsequently enrolled in this study. From the SCMUT database, the median LOS of the inpatients in our unit in the last 5 years was 5.5 days [33]. Therefore, the median LOS measured from our study data was 5.5 days with a range from 2 to 26 days. The study cohort of 108 subjects was divided into two groups according to their length of hospital stay. Patients whose hospital duration of stay was less than 5 days were included in study group A, and patients hospitalized for more than 5 days and over were included in study group B. All procedures performed in the study were conducted according to the ethical standards of the Helsinki declaration. Ethics committee approval was obtained from City Emergency Hospital Timisoara (SCMUT) with the approval number I-27647 on 20 October 2022.

All data were collected per personal data protection rules without personally identifiable data. The outcome variable, the duration of hospitalization (DH), was obtained from the dataset and examined in the current study. DH indicates the number of days that a patient has stayed in the hospital since admission.

The predictor variables were composed of pre-treatment biological inflammatory parameters such as C-reactive protein (CRP), white blood cell count (WBC), with hematological index and neutrophil-to-lymphocyte ratio (NLR). All predictor variables were obtained from the dataset. We also collected demographic data and targeted data such as the site of infection, conditions associated with the potential for immunosuppression, diabetes mellitus status, chronic kidney disease (CKD), and comorbidities that negatively impact current health status (obesity, smoking).

A computer-based medical record was created to proceed with the intended study. The database also contained clinical status, vital signs, and surgical and antibiotic therapy. Descriptive statistics were computed for every study variable. The subdivision of the study group was based on the average duration (in days) of hospitalization, according to the Maxillofacial Department of SCMUT Data. The median hospital stay was 5.5 days.

According to the location of the infection, the site was divided into perimaxillary (vestibular and palatine), perimandibular (vestibular and cutaneous), superficial lodges (submandibular, submental, sublingual, lingual, masseteric, facial), and deep lodges (zygomatic fossa, lateral pharyngeal).

For all patients, a thorough clinical examination was performed on admission. In addition to clinical assessment, the patients underwent routine blood investigations. Furthermore, surgical and antibiotic treatment was applied. Surgical interventions were adjusted to each patient’s condition and included surgical sanitation of the maxillofacial focus, incision, drainage, debridement, extraction, and alveolar curettage. Discharge from the hospital was made when the clinical parameters and laboratory values of the inflammatory markers showed an improved condition, demonstrating the efficacy of the treatment. The main clinical criteria for improvement were body temperature <38 °C, decreased edema or erythema, cessation of trismus and other specific symptoms on admission, and normalization of the values of biological parameters indicative of infection.

### 2.2. Inclusion and Exclusion Criteria

Patients diagnosed with maxillofacial infections from January 2017 to April 2022 at the Department of Maxillofacial Surgery—SCMUT and requiring surgical treatment under general intravenous anesthesia were included in our retrospective study. Each patient was admitted to the hospital for intravenous antibiotic administration and was surgically treated according to operating protocols.

The criteria for patient inclusion were the following:

1. Patients age 18 years and over;
2. Patients with complete medical records;
3. Patients suffering from an abscess of maxillofacial origin, according to the ICD-10 classification of the diseases [39].

The criteria for patient exclusion were the following:

1. Aged under 18 years;
2. Pregnant women;
3. Patients with cancer of different locations;
4. Infections of other regions except for the head and neck;
5. Non-maxillofacial head and neck infection;
6. Incomplete medical records.

### 2.3. Explanatory Variables

The following variables were considered for analysis:

- Demographic data (age, gender, and environment of origin);
- Routine blood test on admission to the hospital, WBC (nr × 10³/μL), CRP (mg/L), and hemogram index NLR—values were obtained by dividing absolute neutrophil and lymphocyte counts;
- The duration of hospitalization (DH) was measured in days starting on the day of the patient’s admission through the Emergency Unit of the Department of Maxillofacial Surgery.

We define the term “faster recovery” as a duration of hospitalization of fewer than 5 days, and the term “prolonged hospitalization” as a duration of hospitalization of ≥5 days, which was the average length of hospitalization.

The patients were discharged from the hospital when they were afebrile and clinically improved after switching from intravenous to oral antibiotics. The main clinical criteria for improvement were body temperature < 38 °C, decreased edema or erythema, and cessation of trismus.

### 2.4. Statistical Analysis

A descriptive analysis was initially performed for all study variables. Continuous variables are presented as mean ± standard deviation (SD) for normally distributed data or as median (interquartile range) for skewed data. Student’s *t*-test was used for the comparison between groups of continuous variables showing normal distribution, and the Mann–Whitney test was used for the comparison of continuous variables not showing normal distribution. The chi-square test or Fisher’s exact test was performed as appropriate for categorical variables comparison. Logistic regression was used to examine the association between DH and CRP, WBC, and NLR. The odds ratio (OD) and its 95% confidence interval (95% CI) were calculated. Sensitivity and specificity after logistic regression to predict dichotomous outcomes were calculated. The receiver operator characteristic (ROC) curve for WBC, CRP, and NLR was plotted and the area under the curve (AUC) was calculated. The cut-off values of the predictor variables were determined from receiver operating characteristic (ROC) analysis using the criterion for determining the cut-off value corresponding to the particular point, where the test Se is equivalent to the test Sp (Se = Sp). The *p* values for all hypothesis tests were two-sided, and statistical significance was set at *p* < 0.05. All analyses were conducted with MedCalc program (MedCalc Software B.V., Ostend, Belgium) and Stata 17 (StataCorp, College Station, TX, USA).

## 3. Results

### 3.1. Demographic Characteristics of the Study Population

Our analysis of the study population included 66 males (61.11%) and 42 females (38.9%). Their mean age was 49.15 years (18.21). The mean age of group A was 44.05 years (age range 18–85), and the mean age of group B was 59.5 years (age range 20–81).

Sixty-two (57.4%) of the patients belonged to urban areas, and the remaining 46 (42.6%) were from rural areas. The urban locality is the one where the majority of labor resources are employed in non-agricultural activities with a diversified level of endowment and equipment, exerting a constant and significant socioeconomic influence on the surrounding area [37]. Comorbidities such as diabetes mellitus and smoking were associated with a prolonged duration of hospitalization among patients with maxillofacial infections. In our study, the most frequent spaces involved were superficial lodges (51.9%), followed by perimandibular space (29.7%), and the least involved space found was deep lodges space (2.8%). The summarized demographic characteristics of the study population are presented in Table 1.

### 3.2. Serum Markers at Admission

The median levels of the biological variables were 11,105 white blood cells/μL (9833–11,700), 63.5 mg/L for CRP (49.67–78.63), and 3.23 per mm^3^ (3.08–3.58) for NLR. The median hospital stay was 5.5 days. The median levels of the studied biological variables and the median duration of hospitalization were significantly higher in Group B. The data are reported in Table 2.

A further linear regression verified the independent effect of the predictor variables (WBC, CRP, and NLR) on the duration of hospitalization, showing a moderate statistically significant positive linear relationship between the two variables (WBC and CRP) and the outcome: duration of hospitalization (*p* = 0.003 and *p* = 0.0002, respectively). The participants’ predicted length of hospital stay was equal to y = 4.0149 + 0.3295 × X *, with WBC measured as white blood cells nr × 10³/μL. This model indicates that each time the WBC increased by 1 white blood cell × 10³/μL, the hospital stay increased by 0.3 days. In other words, when the WBC is equal to 10,000 white blood cells/μL, the predicted hospital stay is 7 days. For CRP, measured as mg/L, the participants’ predicted length of hospital stay was equal to y = 5.4258 + 0.03430 × X *. This model indicates that each time the CRP increased by 1 mg/L, the hospital stay increased by 0.03 days (Table 3). That is to say, when the CRP is equal to 120 mg/L, the predicted hospital stay is 9 days. The results of the association between the duration of hospitalization and the predictor variables from the univariate and multivariable analysis are summarized in Table 3.

In our study, only comorbidities such as diabetes mellitus and smoking were associated with a prolonged duration of hospitalization among patients with maxillofacial infections (Table 1), but were not taken into account in the present study. Furthermore, the coefficient of determination R^2^ for WBC = 0.0786, and the coefficient of determination R^2^ for CRP = 0.1219 showed a moderate statistically significant relationship between the two variables (WBC and CRP) and the outcome: duration of hospitalization. Instead, as shown in Table 3, the logistic regression was used, and WBC (OR 1.239; 95% CI 1.1023 to 1.3933), and CRP (OR 1.013; 95% CI 1.0051 to 1.0227) were found to be statistically significant (*p* < 0.00 and *p* = 0.002, respectively) predictors for the duration of hospitalization among adult patients with maxillofacial infections. A *p*-value < 0.05 was considered statistically significant, and OR with their respective 95% confidence interval was calculated (Table 3).

Furthermore, we performed the ROC curve analysis for the predictor variables (WBC, CRP, and NLR) presented in Table 4.

**Figure 1:**
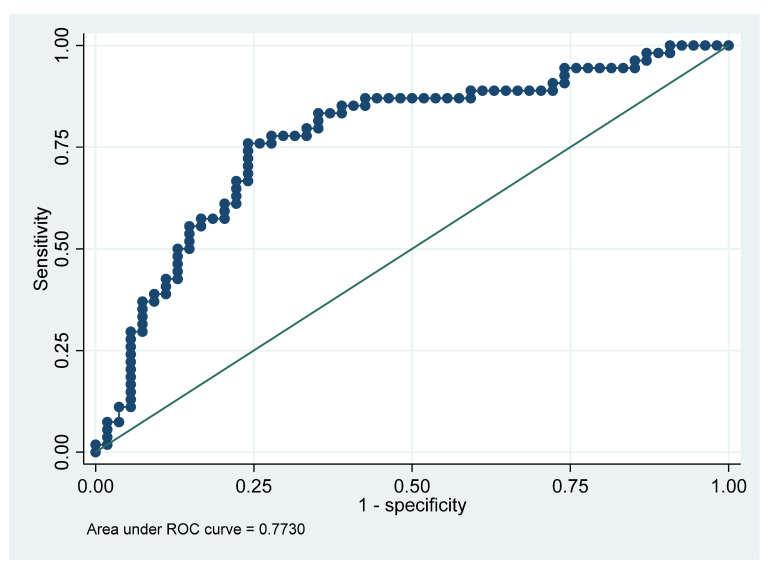
Area under the ROC curve (AUC) for WBC and CRP.

The area under the curve (AUC) identified both the WBC and CRP as accurate predictors for the duration of hospitalization of maxillofacial infections. As described in Table 4, the AUC for the WBC in predicting prolonged hospitalization was 79.6%, compared to the AUC for the CRP of 64.8%. Lastly, the AUC for the WBC, in addition to CRP in predicting prolonged hospitalization, was 77.8%, as seen in Figure 1.

- Number of observations = 108.
- Area under ROC curve = 0.7730.

Furthermore, the cut-off values of the studied predictor variables (WBC and CRP) were determined from the receiver operating characteristic (ROC) analysis (Table 4). We operated one of the frequently used criteria for the determination of the cut-off value as the one corresponding to the particular point, where the test Se is equivalent to the test Sp (Se = Sp) [40,41].

In other words, the cut-off point defined by the Index of Union (IU) method should satisfy two conditions:

1. Sensitivity and specificity obtained at this cut-off point should be simultaneously close to the AUC value;
2. The difference between sensitivity and specificity obtained at this cut-off point should be minimum [41].

**Figure 2:**
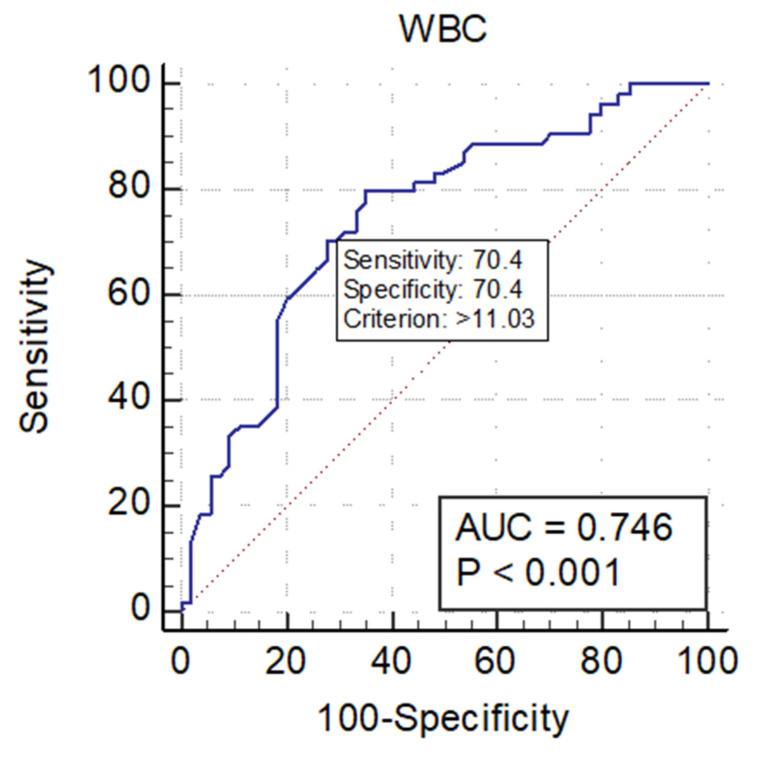
Corresponding point of cut-off values of WBC on the ROC curve (AUC) (Se = Sp).

**Figure 3:**
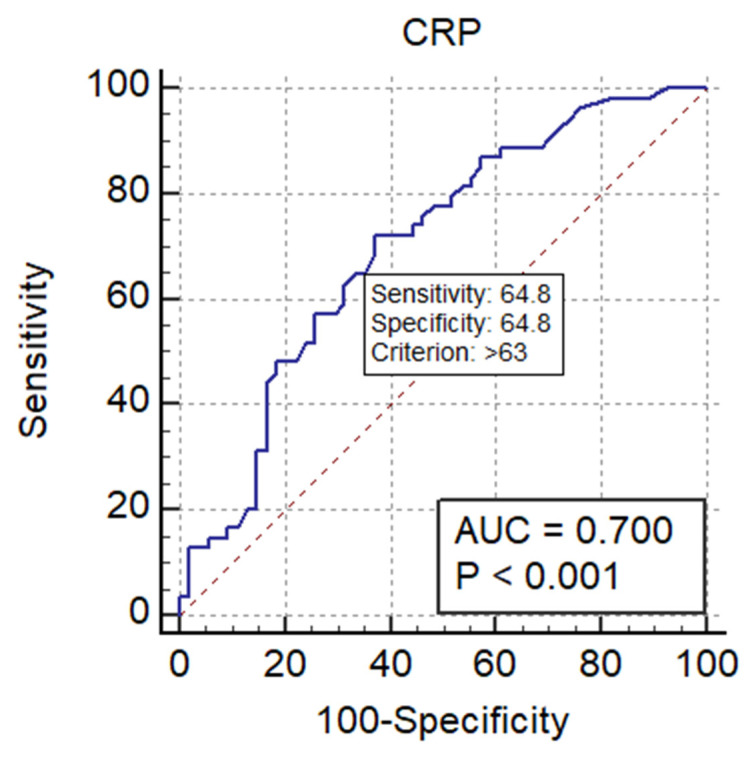
Corresponding point of cut-off values of CRP on the ROC curve (AUC) (Se = Sp).

The pictogram representations for the cut-off values of the study variables (WBC and CRP) from the receiver operating characteristic curve (AUC) analysis are presented in Figure 2 and Figure 3. As shown in the pictograms, the cut-off values determined in our study for the predictor variables are 11,030 white blood cells/μL for WBC, and 63 mg/L for CRP. We found that levels that exceed these cut-off values of the variables WBC and CRP predict a duration of hospitalization of over ≥5 days.

## 4. Discussion

This study was performed to analyze the role of C-reactive protein (CRP), white blood cell count (WBC), and neutrophil-to-lymphocyte ratio (NLR) in predicting the prolonged duration of hospitalization in adult patients with maxillofacial infections. The null hypothesis of this study is that WBC, CRP, and NLR values do not change the variability of the duration of hospital stay.

Our results suggest that WBC and CRP should be used as biological parameters for the prediction of the duration of hospitalization with moderate statistical significance among adult patients with maxillofacial infections.

There have been different results in the literature between prognostic predictors of maxillofacial infection severity and duration of hospital stay. Some studies have identified that WBC count on admission is a predictor of the duration of hospital stay in patients with maxillofacial infections [36,42]. Our study also confirmed this hypothesis. The logistic regression analysis confirmed a positive correlation between WBC count at admission and the duration of hospital stay (*p* < 0.001), and this had the highest specificity (64.81%) from the studied variables.

Ylijoki et al. [43] concluded that even though the white blood cell (WBC) count and the erythrocyte sedimentation rate (ESR) help to define the state of the patient on admission, their prognostic predictability is limited. Additionally, the literature has shown serum CRP at admission or preoperatively as a significant predictive factor for length of hospital stay in patients with maxillofacial infections [44,45]. Most of these studies have found that CRP was an effective parameter for predicting hospital length of stay [28]. Similarly, using a quantitative approach, Sharma et al. [46] showed that the CRP level was a significant marker of the length of hospital stay. The higher the CRP concentration, the longer the patient’s hospital stay. Our study confirmed a statistically significant positive correlation between CRP levels at admission and the prolonged duration of hospital stay, and a significant difference between groups. We showed that admission CRP was strongly correlated with the duration of hospital stay (*p* < 0.001) and may predict the duration of stay in patients with maxillofacial infections of maxillofacial origin with moderate sensitivity (72.22%) and specificity (62.96%).

Furthermore, we chose criteria to find the optimal cut-off values of the studied biological markers for predicting the duration of hospitalization. The choice of criteria in determining the optimal cut-off value is a matter of concern in quantitative diagnostic tests. Several indexes, such as the Index of Union (IU), Youden’s index, Euclidean index, and product of sensitivity and specificity in receiver operator characteristic space have been used in clinical practice. Still, their advantages and limitations are not well understood by clinicians [40]. We operated one of the frequently used criteria for the determination of the test cut-off value as the one corresponding to the particular point, where the test Se is equivalent to the test Sp (Se = Sp) [40,41]. The cut-off values of the studied predictor variables (WBC and CRP) determined from the receiver operating characteristic (ROC) analysis were 11,030 white blood cells/μL for WBC, and 63 mg/L for CRP. We found that levels that exceed these cut-off values of the variables WBC and CRP predict a duration of hospitalization of over ≥5 days.

The inflammatory response in the body during bacterial infections is characterized by neutrophilia and lymphocytopenia. This relationship between inflammatory cells is verified by the neutrophil-to-lymphocyte ratio, an efficient marker used to diagnose bacterial infections [47]. Dogruel et al. [48] demonstrated the effectiveness of the neutrophil-to-lymphocyte ratio as a prognostic marker of maxillofacial infections and its correlation with the length of hospital stay. Regarding laboratory tests of the values of leukocytes, neutrophils, and NLR, Ishimine et al. [49] showed a significant difference between the groups and a positive and statistically significant correlation regarding the length of hospitalization. The greater the severity of the case, the greater the values will be in the laboratory tests and, consequently, the longer the hospital stay. In a study of 1468 patients with suspected bacteremia and septicemia, using procalcitonin (PCT) as a reference, Gürol et al. [50] found NLR to have higher sensitivity than CRP and WBC. Instead, our study found the lowest specificity and sensitivity for NLR values in predicting the prolonged duration of hospital stay. In patients with maxillofacial space infection, a prolonged hospital stay is associated with substantial financial costs to health care services. The average cost per night for a bed in SCMUT hospital is USD 197.69 (RON 900). When day surgeries were added, the average cost per patient with maxillofacial infection was USD 389.28 (RON 1773). The reduction in LOS from 3 days to 1 day effectively cut the inpatient cost by 33.3% [51].

In our study, only comorbidities such as diabetes mellitus and smoking were associated with a prolonged duration of hospitalization among patients with maxillofacial infections.

Several other studies reported that the LOS of patients admitted for maxillofacial infections was significantly prolonged in those with pre-existing medical conditions [30] such as poorly controlled diabetes mellitus and hypertension, which required longer LOS for stabilization.

In another study [28] based on a retrospective chart review of severe odontogenic infections in 298 patients admitted for the treatment of severe odontogenic infections at three hospitals in Houston, TX (Ben Taub, Memorial Hermann Hospital, and Lyndon B. Johnson) from January 2010 through January 2015, the authors identified that age, preadmission antibiotic use, diabetes mellitus, and higher odontogenic infection severity score were associated with increased LOS.

It is therefore important to determine whether various serum inflammatory marker levels on admission may predict a prolonged hospital stay, thus facilitating more efficient bed management. In addition, economically, the inflammatory markers evaluated in our study should become good predictors for the duration of hospitalization.

## 5. Limitations of the Study

The limitations of this study included its retrospective nature, which means it is dependent on medical records data, being more prone to human error, and a relatively small sample size of patients, which requires further validation in the future. Limited by the retrospective design of our study, we could not perform a dynamic profile analysis of WBC, CRP, and NLR, which may offer more helpful information. Further studies with longer monitoring and a broader sampling of patients would address these limitations so that the results can be generalized to the local population.

The inability to identify the impact of pre-existing immunocompromised status on the severity of the infection and, subsequently, on the duration of hospitalization because of our relatively small sample of patients with a history of immunosuppression is also a limiting factor.

## 6. Conclusions

Within the limitations of this study, our results have shown a moderate statistically significant positive correlation between the levels of predictor variables (WBC count, C-reactive protein) on admission and the duration of hospitalization. These results indicate that serum WBC count and CRP levels on admission should be used as biological parameters for the prediction of the duration of hospitalization in adult patients with maxillofacial infections.

The optimal cut-off values determined in our study for the predictor variables are 11,030 white blood cells/μL for WBC, and 63 mg/L for CRP. Levels that exceed these optimal threshold values for the variables WBC and CRP predict a duration of hospitalization of over (≥) 5 days.

The participants’ predicted length of hospital stay indicates that each time the WBC increased by 1 white blood cell × 10³/μL, the hospital stay increased by 0.3 days, and each time the CRP increased by 1 mg/L, the hospital stay increased by 0.03 days.

Among the assessed parameters, WBC count was confirmed as the most sensitive indicator for the duration of hospitalization in adult patients with maxillofacial infections.

Given that the duration of hospitalization is a key determinant of the financial cost of hospital care, the ability to predict it using simple and inexpensive biological parameters (such as CRP and WBC count) may improve more cost-effective care and efficient hospital bed management.

## Figures and Tables

**Table 1:** Patients’ characteristics.

Variables	Short Hospitalization (Group A) (*n* = 54)	Long Hospitalization (Group B) (*n* = 54)	Significance
Age, mean (SD) *	44.05 (19.17)	59.5 (15.79)	0.047 *
Age range	18–85	20–81	-
Gender, *n* (%) Men ***	34 (62.96%)	32 (59.25%)	0.843 ***
Gender, *n* (%) Women ***	20 (37.03%)	22 (40.74%)	
Place of origin, *n* (%) Urban ***	32 (59.25%)	30 (55.55%)	0.845 ***
Place of origin, *n* (%) Rural ***	22 (40.74%)	24 (44.44%)	
Smoking, *n* (%) Yes ***	9 (8.3)	19 (17.6)	0.047 ***
Smoking, *n* (%) No ***	45 (32.4)	35 (17.6)	
Comorbidities, *n* (%) Diabetes **	10 (9.3)	28 (25.9)	<0.001 **
Comorbidities, *n* (%) Obesity **	31 (28.7)	37 (34.3)	0.466 **
Comorbidities, *n* (%) Chronic kidney disease **	14 (13.0)	17 (15.7)	0.590 **
Comorbidities, *n* (%) Human Immunodeficiency Virus **	1 (0.9)	1 (0.9)	1.000 **
Comorbidities, *n* (%) Malignancy **	5 (4.6)	7 (6.5)	0.563 **
Location of the infection site, *n* (%) Perimaxillary **	13 (12.0)	10 (9.3)	0.531 **
Location of the infection site, *n* (%) ** Perimandibular	14 (13.0)	18 (1.7)	0.479 **
Location of the infection site, *n* (%) Superficial lodges **	27 (25.0)	29 (26.9)	0.789 **

Notes: * Student’s *t*-test; ** chi-square test; *** Fisher’s exact test. Abbreviation: SD, standard deviation.

**Table 2:** Study markers and duration of hospitalization.

Variables	Total Subjects		A (Short Duration of Hospitalization)		B (Long Duration of Hospitalization)		*p*-Value *
	Median *	95% CI *	Median *	95% CI *	Median *	95% CI *	
WBC	11,105	(9833–11,700)	9.34	8.240 to 9.900	12.02	11.516 to 12.639	*p* < 0.001
CRP	63.5	(49.6–78.63)	45.95	19.660 to 61.348	86.00	65.660 to 98.000	*p* < 0.001
NLR	3.23	(3.08–3.58)	2.99	2.34 to 3.29	3.51	3.20 to 3.88	*p* = 0.038
Duration of hospitalization	5.5	(5.0 to 7.66)	4.0	3.00 to 4.00	10.0	9.00 to 12.66	*p* < 0.001

Notes: * Data reported were calculated using the Mann–Whitney test. Abbreviations: CI, confidence interval; WBC, white blood cell count; CRP, C-reactive protein; NLR, neutrophil-to-lymphocyte ratio.

**Table 3:** Univariate and multivariate analysis for predictor variables.

Linear Regression for Predictor Variables	WBC		CRP		NLR	
Coefficient of determination R^2^	0.0786		0.1219		0.0086	
Residual standard deviation	5.2600		5.1349		5.4560	
	y = 4.0149 + 0.3295 × X *		y = 5.4258 + 0.0343 × X *		y = 7.760 + 0.0057 × X *	
	Coeff.	SE.	Coeff.	SE.	Coeff.	SE.
Intercept	4.0149	1.3670	5.4258	0.7987	7.7609	0.5304
Slope	0.3295	0.1096	0.0343	0.0089	0.0057	0.0059
F-ratio	9.0426		14.7197		0.9280	
Significance level	*p* = 0.003		*p* = 0.0002		*p* = 0.337	
Correlation coefficient r	0.2804		0.3492		0.0931	
Logistic regression for predictor variables	WBC		CRP		NLR	
Odds ratio	1.2394		1.0139		1.1097	
95% CI	1.1023 to 1.3933		1.0051 to 1.0227		0.9673 to 1.2730	
*p*-value	*p* < 0.001		*p* = 0.002		*p* = 0.137	
Multiple regression for predictor variables	WBC		CRP			
Odds ratio	1.1992		1.0099			
95% CI	1.0659 to 1.3491		1.0011 to 1.0189			
*p*-value	0.003		0.027			

Notes: * Linear regression equation (y = b0 + b1 × X) where b0 is a constant (intercept), b1 is the regression coefficient, X is the value of the independent variable, and y is the predicted value of the dependent variable. Abbreviations: WBC, white blood cell count; CRP, C-reactive protein; NLR, neutrophil-to-lymphocyte ratio; Coeff., coefficient; SE., standard error; CI, confidence interval.

**Table 4:** ROC curve analysis for study variables and the cut-off values for WBC and CRP.

Variables	Cut-Off Values	Area under ROC Curve (AUC)	95% CI	Significance Level *p*	Sensitivity	Specificity
WBC	11.03 *	0.746	0.653 to 0.825	<0.001	79.63	64.81
CRP	63 **	0.700	0.605 to 0.785	<0.001	72.22	62.96
NLR		0.616	0.517 to 0.708	0.037	75.93	57.41
WBC and CRP		0.773		<0.001	66.67%	77.78%

Notes: * white blood cell/per microliter (nr × 10³/μL); ** mg/L. Abbreviations: ROC, receiver operating characteristic curve; AUC, area under the curve; CI, confidence interval; WBC, white blood cell count; CRP, C-reactive protein; NLR, neutrophil-to-lymphocyte ratio.

## Data Availability

Data available on request.
